# Functional connectivity dynamics of monoaminergic circuits related to fatigue in multiple sclerosis

**DOI:** 10.1007/s00415-026-13876-0

**Published:** 2026-06-02

**Authors:** Marloes D. A. Bet, Birgit Helmlinger, Tommy A. A. Broeders, Stefanie M. C. Hechenberger, Christian G. Tinauer, Stefan Ropele, Frederik Barkhof, Michael Khalil, Christian Enzinger, Linda Douw, Menno M. Schoonheim, Daniela T. Pinter

**Affiliations:** 1 MS Center Amsterdam, Anatomy and Neurosciences, Vrije Universiteit Amsterdam, Amsterdam Neuroscience, Amsterdam UMC Location VUmc, Amsterdam, The Netherlands; 2 Department of Neurology, Medical University of Graz, Graz, Austria; 3 Department of Oncology, Medical University of Graz, Graz, Austria; 4 MS Center Amsterdam, Radiology and Nuclear Medicine, Vrije Universiteit Amsterdam, Amsterdam Neuroscience, Amsterdam UMC Location VUmc, Amsterdam, The Netherlands; 5 Queen Square Institute of Neurology and UCL Hawkes Institute, University College London, London, UK; 6 Department of Anatomy and Neurosciences, Amsterdam Neuroscience, Cancer Center Amsterdam, Amsterdam University Medical Centers Location Vrije Universiteit Amsterdam, Amsterdam, The Netherlands; 7 Amsterdam Neuroscience, Systems & Network Neuroscience, Amsterdam, The Netherlands

**Keywords:** Fatigue, Multiple sclerosis, Functional MRI, Networks, Monoamines

## Abstract

**Introduction:**

Monoaminergic neurotransmitters help regulate the interplay between the central nervous system and the immune system. Aberrant monoaminergic functional connectivity is presumably involved in MS-related fatigue, but mechanisms remain unclear.

**Aim:**

To investigate how static and dynamic functional connectivity of monoaminergic circuits relate to fatigue in MS.

**Methods:**

60 healthy controls (HC) and 217 people with MS (pwMS) completed the Fatigue Scale for Motor and Cognitive Functions (FSMC) and underwent functional brain MRI. Brain regions were assigned to resting-state networks. Additionally, PET-derived receptor/transporter density atlases determined assignment to monoaminergic circuits (5-HT_1a_/5-HT_2a_/5-HTT/ D_1_/D_2_/DAT/NAT). Per monoaminergic circuit, we compared the frequency of switching between networks (flexibility) and the total number of networks switched to (promiscuity), between HC and pwMS with no fatigue (FSMC < 43), mild/moderate fatigue (43 ≤ FSMC < 63), or severe fatigue (FSMC ≥ 63). For circuits showing flexibility differences, we also compared rates of synchronous (cohesion) and independent switches (disjointedness).

**Results:**

Global flexibility was higher in severely fatigued pwMS compared to non-fatigued pwMS (*p* = 0.025) and HC (*p* = 0.016). In line, global cohesion was higher in severely fatigued pwMS compared to non-fatigued pwMS (*p* = 0.022) and HC (*p* = 0.017). Compared to HC, 5-HT_1a_ circuit flexibility (*p* = 0.048) and cohesion (*p* = 0.031) were higher in severely fatigued pwMS. Static FC showed significant group differences for the 5-HTT, D_1_, and D_2/_DAT circuits, but no significant pairwise contrasts.

**Conclusion:**

Fatigued pwMS show more dynamic functional connectivity, both globally and in the inhibitory serotonergic receptor circuit. Unstable global and serotonergic dynamics likely raise energy expenditure in pwMS and therefore may underlie MS-related fatigue.

**Supplementary Information:**

The online version contains supplementary material available at 10.1007/s00415-026-13876-0.

## Introduction

Fatigue occurs in up to 95% of people with multiple sclerosis (pwMS) [1], and is associated with worse work-related outcomes and quality of life [2, 3]. Evidence on pathophysiological mechanisms underlying MS-related fatigue is scarce and remains inconclusive. Since monoaminergic neurotransmitters can modulate the bidirectional interplay between the central nervous system and the immune system by acting on receptors of both systems, they might be implicated in MS pathology [4]. While monoamines promote immunosuppression, existing inflammation *inhibits* monoamine synthesis, thus dampening monoaminergic immunosuppression and prompting a vicious cycle of inflammation [4].

Additionally, MS-related fatigue is hypothesized to arise partly through atypical network recruitment [5]. Indeed, network evidence suggests that aberrations of *static* functional connectivity (FC) in monoaminergic circuits (serotonin [5-HT], dopamine [DA], and noradrenalin [NA]) underlie fatigue in pwMS [6, 7]. For instance, MS-related fatigue has been linked to lower connectivity in the dopamine network (i.e. left thalamus and cerebellum) and increased connectivity in the serotonin network (i.e. occipital gyrus) [6], as well as regional alterations in DA and NA transporter connectivity. [7] However, these studies have only considered regions linked to monoaminergic *transporters* and not to receptors, which are more directly involved in neurotransmission, and therefore are of indispensable importance in determining patterns of functional connectivity [8].

Furthermore, beyond static functional connectivity (FC), the dynamics of monoaminergic circuits may also be relevant for MS-related fatigue [9]. Indeed, FC fluctuations have been linked to many different brain functions and may be adaptive or maladaptive in different contexts [10]. In pwMS, local dynamics have been shown to be more chaotic, characterized by more connectivity switching and a higher variation in connectivity partners [11]. This may be of particular interest for MS-related fatigue, as an fMRI study showed that in pwMS, brain regions that re-allocate connectivity from one network to another consume more metabolic energy while doing so [12]. Thus, on top of atypical network recruitment [5], a higher degree of switching and variation in connectivity pairs might be more energy-consuming and thus contribute to the subjective experience of fatigue.

Therefore, in this study, we aimed to investigate whether FC of monoaminergic circuits is related to fatigue in MS, using both static and dynamic approaches. We hypothesize that more chaotic dynamics of FC, specifically in monoaminergic circuits, may contribute to MS-related fatigue. Further, static FC may be generally lower because MS-related pathology impairs signal transduction along WM tracts. Both increases and decreases of monoaminergic static FC have been reported using different analytical approaches and different regions of interest [7, 13].

Further, fatigue can be subdivided into physical (motor) and mental (cognitive) fatigue [14]. Evidence on distinct mechanisms of cognitive vs. motor fatigue in pwMS remains scarce. Thus, as a secondary exploratory objective, we aim to assess if dynamics of the three monoaminergic networks are distinctly associated with cognitive fatigue or motor fatigue. Cognitive fatigue has been related to FC abnormalities in frontal NA transporter connectivity [15] and dopaminergic circuits involved in interoceptive processing and reward pathways [16, 17]. Also, motor fatigue has been related to depression and anxiety in early MS, possibly implying a role for monoaminergic signaling [18].

## Methods

### Participants and study procedures

Study procedures were approved by the ethical review board of the Medical University of Graz (31–432 ex 18/19 1264–2019). All participants provided written informed consent prior to inclusion.

We recruited 241 pwMS and 87 healthy controls (HC) from the Graz resting-state functional magnetic resonance imaging (rs-fMRI) MS cohort at the Department of Neurology of the Medical University of Graz. Participants underwent neuropsychological assessment and magnetic resonance imaging of the brain.

For pwMS, we collected MS phenotype (clinically isolated syndrome, relapsing–remitting, or progressive) and disease duration from first symptom onset. A certified examiner estimated the Expanded Disability Status Scale (EDSS) score. Information on high vs. low-efficacy treatment, based on current standards of disease-modifying therapies (DMT) approved for MS treatment in Germany and Austria, was recorded [19]. PwMS who switched DMTs within 8 weeks of participation in the study were excluded.

**Fig. 1:**
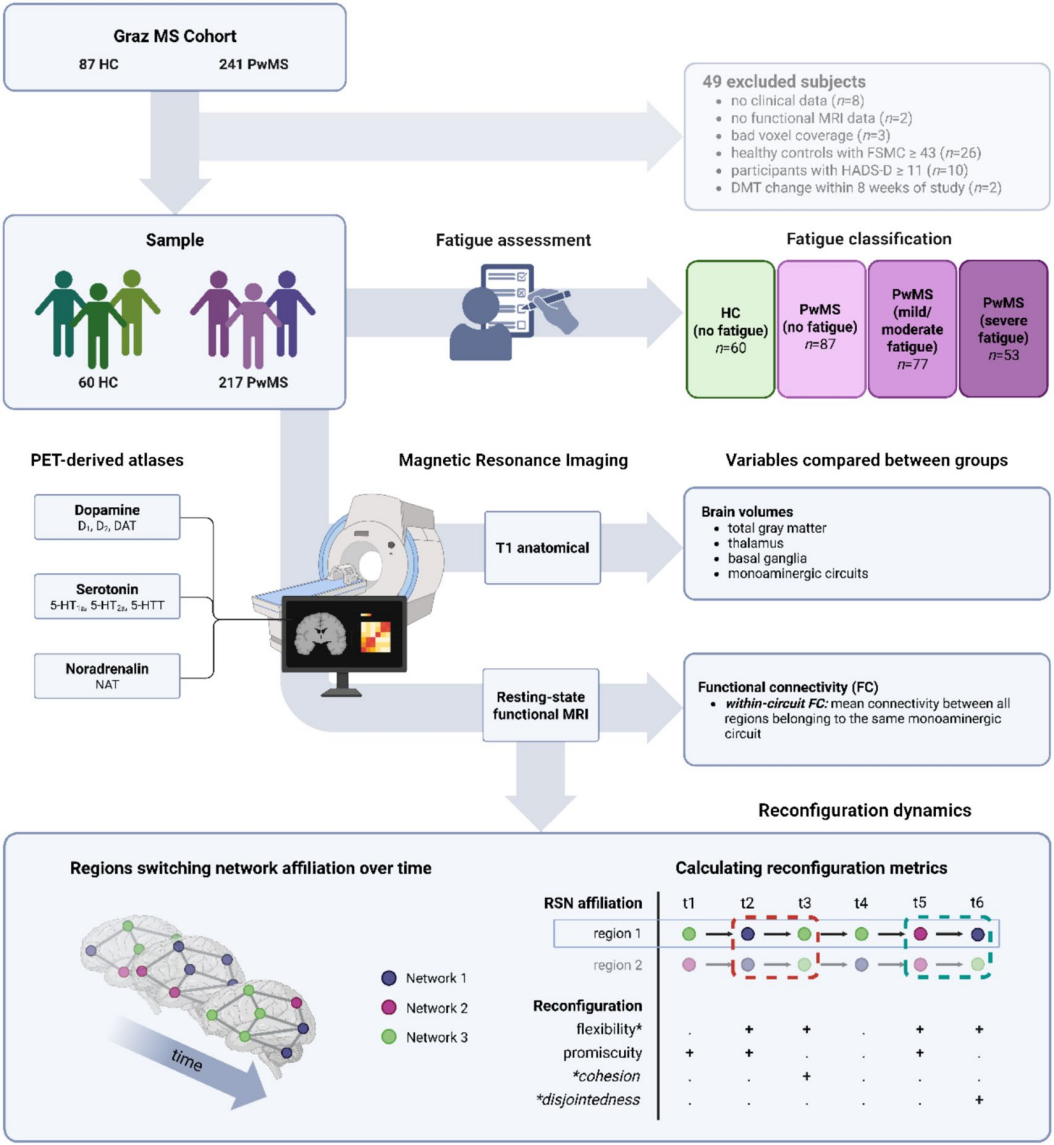
Study procedures. Based on fatigue score, people with MS were classified as having no fatigue, mild/moderate fatigue, or severe fatigue. Healthy controls reporting fatigue were excluded from analysis. Based on normative PET-derived atlases of monoaminergic receptor and transporter expression, we delineated serotonergic, dopaminergic, and noradrenergic circuits, for which we calculated brain volumes, measures of functional connectivity, and reconfiguration dynamics. Since cohesion and disjointedness depend on the occurrence of a switch, we only compared them between groups if significant group differences in flexibility were observed. *MS* multiple sclerosis, *FSMC* Fatigue Scale for Motor and Cognitive Function, *HADS-D* Hospital Anxiety and Depression Scale—Depression Subscale, *DMT* disease-modifying treatment, *HC* healthy controls, *PET* positron emission tomography, *D*_*1*_ dopamine-1 receptor, *D*_*2*_ dopamine-2 receptor, *DAT* dopamine transporter, *5-HT*_*1a*_ serotonin-1a receptor, *5-HT*_*2a*_ serotonin-2a receptor, *5-HTT* serotonin transporter, *NAT* noradrenalin transporter, *MRI* magnetic resonance imaging, *ROI* regions of interest, *RSN* resting-state network

For all participants, we used the Fatigue Scale for Motor and Cognitive Functions (FSMC) [14] to assess the severity of total, cognitive, and motor fatigue. Items were answered on a 5-point Likert scale with a maximum score of 100. Mild, moderate and severe fatigue were indicated by scores of at least 43, 53, and 63 for total fatigue; 22, 28, and 34 for cognitive fatigue; and 22, 27, and 32 for motor fatigue [14]. Healthy controls reporting mild, moderate or severe fatigue were excluded (Fig. 1).

To distinguish fatigue from anxiety and depression [20, 21], we administered the Hospital Anxiety and Depression Scale (HADS) [22]. Given the high comorbidity and pathophysiological overlap between fatigue and depression, participants with scores ≥ 11, signifying moderate (clinically relevant) depression, were excluded.

### Magnetic resonance imaging

#### Acquisition parameters

Participants were scanned on a Siemens MAGNETOM 3 T Prisma Fit system with a 20-channel head coil at the Medical University of Graz, Austria. The protocol included a 3D-T1 MPRAGE sequence (TR = 1900 ms, TE = 2.7 ms, 1 mm isotropic resolution); a 3D-FLAIR sequence (TR = 5000 ms; TE = 393 ms, TI = 1800 ms, 1 mm isotropic resolution); and an eyes-closed single-shot multi-band (factor 4) echoplanar resting-state functional MRI sequence (300 volumes, 52 slices, TR = 1000 ms, TE = 35 ms, field of view 256 × 256 mm^2^, acquisition time 5.20 min, 2 mm isotropic resolution).

#### Preprocessing

After reorienting to standard space, we applied SPM’s lesion prediction algorithm to FLAIR images, inspected and corrected the resulting lesion masks, and filled the T1-weighted images using an FSL-based pipeline (https://github.com/neuroimaging-mug/ms-volest). We used FSL’s (v6.0) SienaX to segment GM, WM, and CSF, and FSL-FIRST to more reliably segment volumes of deep GM regions.

Using SyNBOLD-DisCo, we generated artificial field maps and performed distortion correction and motion correction on the functional images. Using FSL-FEAT, we performed linear registration (FSL-FLIRT) of fMRI images to T1 space, non-linear registration (FSL-FNIRT) to MNI152-standard space, and 4 mm spatial smoothing. ICA-AROMA removed physiological noise components and calculated motion parameters, which were regressed out of the signal. To detect signal originating from WM, the SienaX-derived WM mask was linearly registered to functional space. To detect signal originating from CSF, the MNI152 standard ventricle mask was non-linearly registered to native anatomical space, multiplied with the inverse of the FIRST-derived deep GM mask and the SienaX-derived CSF mask, and linearly registered to functional space. Time series and derivatives of WM and CSF signal were then regressed out of the signal, and high-pass filtering was applied to the resulting BOLD time series (sigma = 100/2*TR).

To delineate brain regions in subject space, we combined the 210 cortical parcellations from the Brainnetome atlas (BNA), registered to native anatomical space using the inverse registration matrix produced by FSL-FNIRT, with 14 deep GM regions delineated by FSL-FIRST. Due to the limited field of view in many functional images, the cerebellum was excluded. Participants for whom > 12 brain regions had zero voxel coverage (*n* = 3) were excluded. Thereafter, 22 regions yielded < 30% voxel coverage in > 10% of participants and were also excluded, yielding 202 regions in total. Region-averaged BOLD time series were correlated and the resulting FC matrices were normalized for maximum connectivity, *r*-to-*z* transformed, and made absolute.

#### Region selection

We delineated monoaminergic circuits using PET/SPECT-derived HC-based maps of 5-HT_1a_ (major inhibitory serotonergic receptor) [23], 5-HT_2a_ (major excitatory serotonergic receptor) [24], inhibitory D_1_-type receptors [25], excitatory D_2_-type receptors [26], and presynaptic transporters DAT [27], 5-HTT [24], and NAT [28] (Table S1), obtained through https://github.com/netneurolab/neuromaps.

**Fig. 2:**
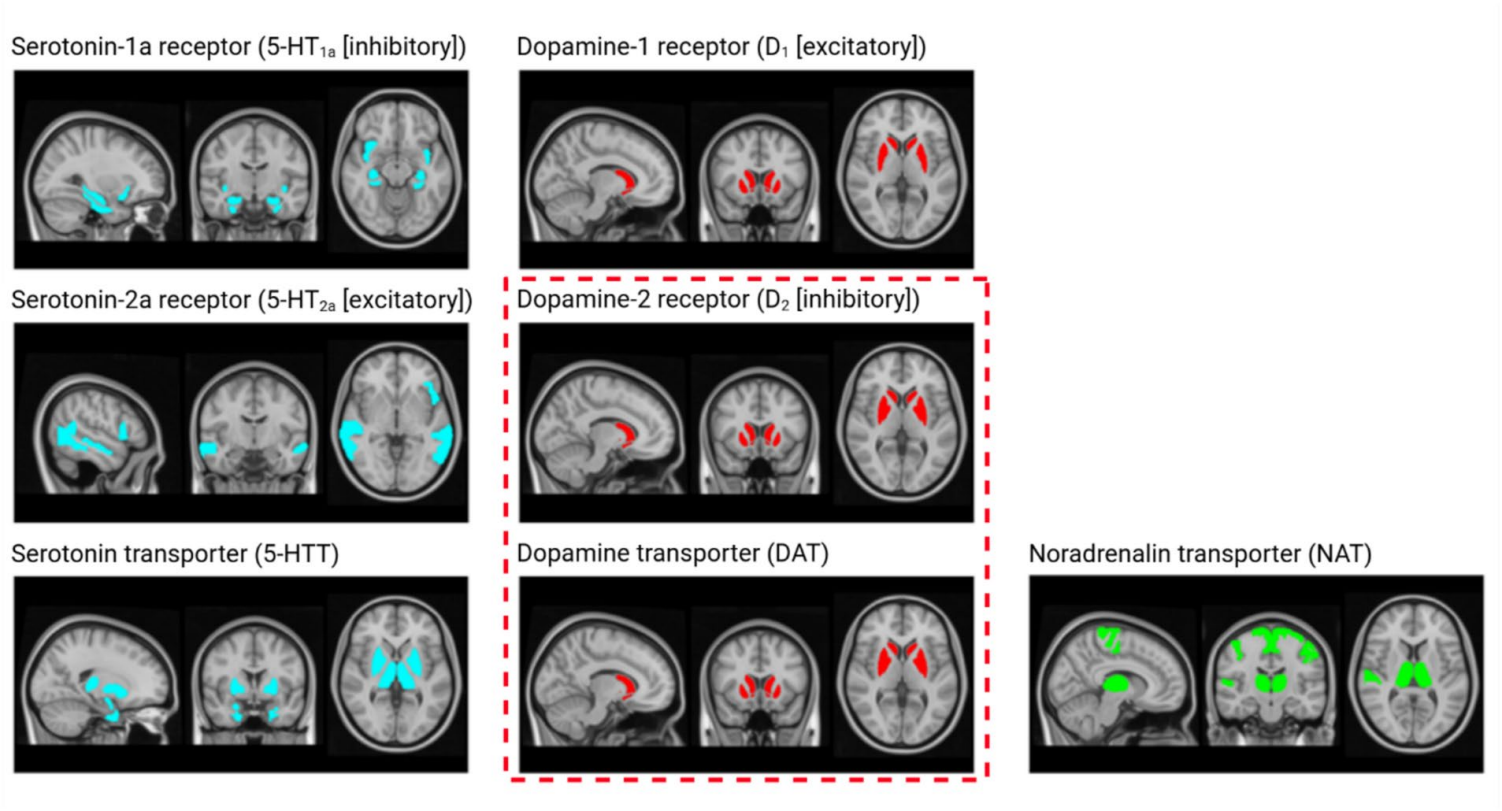
Atlas maps of regions included per monoaminergic receptor/transporter circuit. Red dashed line: Since the same regions showed the highest expression of the D_2_ receptor and dopamine transporter (DAT), results for these receptor/transporter circuits were reported jointly

We created a standard atlas for deep GM (DGM) regions by registering FIRST segmentations from subject-native space to MNI152-standard space and retaining voxels denoting DGM regions in ≥ 50% of HC. After combining this standard FIRST atlas with the BNA, we superimposed the resulting atlas on PET-derived maps to calculate mean receptor/transporter densities per region. Regional densities were normalized at maximum density for cross-map comparability. We used elbow plots to assign the highest-density regions to monoaminergic receptor/transporter circuits, using the “elbow” as the cut-off. For linear slopes, we selected the top 5% (12 regions). Due to full overlap of the D_2_ and DAT circuits, they were reported jointly. Figure 2 and Table S2 show the regions assigned to receptor/transporter circuits.

#### Volumes and lesion load

Since thalamic volume strongly predicts general disease worsening in MS, and damage to the basal ganglia is predictive for MS-related fatigue [29], we calculated thalamic and basal ganglia volumes in addition to total cortical GM and whole-brain lesion load. Further, we calculated GM volumes within the monoaminergic receptor/transporter circuits.

#### Static functional connectivity

By applying Pearson’s correlation across the entire time series, functional connectivity matrices were computed. From these matrices, we extracted within-circuit FC by taking the mean of all FC values between all regions of each monoaminergic circuit.

#### Reconfiguration dynamics

In order to calculate dynamic reconfiguration variables, the BOLD signal was split into 274 overlapping windows of 27 volumes, with step size 1 [30]. FC matrices were constructed per window based on Pearson’s correlation and were *r*-to-*z* transformed and made absolute. Alongside a region-wise parcellation of seven cortical RSNs [31] and DGM [11], these window-for-window FC matrices were used to determine region-wise RSN assignment, i.e. which region was most connected to which RSN in each specific time window (https://github.com/taabroeders/Recon_Dyn_MS). Then, window-for-window RSN assignments were used as input to calculate reconfiguration variables using the openly available Dynamic Graph Metrics toolbox.[32] First, we calculated *flexibility* and *promiscuity* per individual region [11, 32]. *Flexibility* reflected the number of times that a region switched RSNs [10, 33], while *promiscuity* reflected the total number of RSNs switched to. We averaged both measures per monoaminergic circuit, thereby quantifying each circuit’s dynamic properties. In case of group differences in flexibility either globally or in a specific monoaminergic circuit, we calculated *cohesion* and *disjointedness* as well for that same set of brain regions. *Cohesion* reflected regions’ tendency to switch to the same RSN simultaneously with other region(s), while *disjointedness* reflected regions’ tendency to switch RSNs independently (Fig. 1) [34].

## Statistics

### Main analyses

To ensure adequate sample distribution, pwMS were classified in groups characterized by no fatigue (FSMC < 43), mild/moderate fatigue (43 ≤ FSMC < 63), and severe fatigue (FSMC ≥ 63). Between non-fatigued HC, non-fatigued pwMS, mildly/moderately fatigued pwMS, and severely fatigued pwMS, we compared thalamic, basal ganglia, and cortical GM volumes; within-circuit FC; and flexibility and promiscuity per monoaminergic circuit. Since cohesion and disjointedness provided information about the nature of switches, they were only analyzed if group differences in switching frequency (flexibility) existed. We performed all comparisons using ANCOVA (*aov* function in *R* v4.4.3), corrected for sex, age, and years of education. We applied circuit-level Benjamini–Hochberg correction for multiple comparisons.

### Post-hoc analyses

As a sensitivity analysis, we repeated our main analyses on a subsample of relapsing–remitting pwMS only.

In case of significant group differences in the main analyses based on total fatigue, we conducted post-hoc analyses to ascertain whether this variable was specifically related to either motor fatigue or cognitive fatigue. In such, we re-categorized pwMS as non-fatigued, mildly/moderately fatigued, and severely fatigued based on the aforementioned recommended cut-offs for cognitive and motor fatigue [14].

## Results

### Sample

The final sample included 217 pwMS (59.4% female, 39.58 ± 10.03 years of age) and 60 HC (56.7% female, 30.46 ± 8.34 years of age; Fig. 1). Of the included pwMS, 68 (31.3%) were on dimethylfumarate, 19 (8.8%) on ocrelizumab, 16 (7.4%) on interferon-beta, 14 (6.5%) on glatiramer acetate, 12 (5.5%) on fingolimod, 10 (4.6%) on teriflunomide, 5 (2.3%) on natalizumab, 5 (2.3%) on ozanimod, 1 on cladribine (0.5%) and 22 (10.1%) on other types of medication. 45 pwMS (20.7%) were unmedicated at the time of MRI acquisition. Thus, 43 (19.8%) of pwMS were on high-efficacy DMTs, 129 (59.4%) on low-efficacy DMTs, and 45 (20.7%) were not on any DMT.

Table 1 shows sample descriptives. Scores for total, cognitive, and motor fatigue, and anxiety and depression, were higher in pwMS than in HC (all *p* ≤ 0.001). Among pwMS, 87 (40.1%) were non-fatigued (FSMC_*total*_ < 43), 32 (14.7%) were mildly fatigued (FSMC_*total*_ ≥ 43), 45 (20.7%) were moderately fatigued (FSMC_*total*_ ≥ 53), and 53 (24.4%) were severely fatigued (FSMC_*total*_ ≥ 63). Both anxiety and depression scores were higher in severely fatigued pwMS and mildly/moderately fatigued pwMS than in non-fatigued pwMS and HC (all *p* < 0.05).

**Table 1:** Sample characteristics per group

Variable	HC (*n* = 60)	Total pwMS (*n* = 217)	*p*	Non-fatigued pwMS (*n* = 87)	Mildly/moderately fatigued pwMS (*n* = 77)	Severely fatigued pwMS (*n* = 53)	*p*^*a*^
Sex (F/M (%))	34/26 (56.7/43.3)	129/88 (59.4/40.6)	0.811	47/40 (54/46)	17/15 (53.1/46.9)	30/15 (66.7/33.3)	0.329
Age (years)	30.46 ± 8.34	39.58 ± 10.03	** < *0.001***	38.06 ± 8.97	40.59 ± 11.3	40.74 ± 11.07	0.252
Education (years)	17.41 ± 2.98	14.68 ± 3.4	** < *0.001***	15.17 ± 3.34	14.48 ± 3.63	14.08 ± 3.3	0.197
MS type (%, CIS/RRMS/PMS)	*NA*	7/194/16 (3.2/89.4/7.4)	*NA*	1/85/1 (1.1/97.7/1.1)	3/65/9 (3.9/84.4/11.7)	3/44/6 (5.7/83.0/11.3)	***0.027***
EDSS (median (IQR))	*NA*	1 [0–2]	*NA*	1 [0–1.5]	1 [0–2.5]	2 [1–2.5]	** < *0.001***^***e,f***^
Disease duration from onset (years)	*NA*	10.52 ± 7.99	*NA*	8.95 ± 6.46	12.22 ± 9.12	10.62 ± 8.15	***0.032***^***e***^
Treatment (high-efficacy/low-efficacy/none)	*NA*	43/129/45 (19.8/59.4/20.7)	*NA*	11/55/21 (12.6/63.2/24.1)	6/20/6 (18.8/62.5/18.8)	11/26/8 (24.4/57.8/17.8)	0.215
Lesion load (median (IQR))	*NA*	3.91 [1.73–9.87]	*NA*	3.05 [1.67–8.17]	3.20 [1.24–10.03]	4.07 [1.73–7.92]	***0.037***^***f***^
FSMC total fatigue (median (IQR))	29 [25–37.25]	50 [36–62]	** < *0.001***	34 [27–39]	54 [49–59]	74 [68–79]	** < *0.001***
FSMC cognitive fatigue (median (IQR))	16 [13–19]	24 [17–31]	** < *0.001***	16 [12–19]	27 [23–29]	37 [33–40]	** < *0.001***
FSMC motor fatigue (median (IQR))	14.5 [11.75–17]	25 [18–32]	** < *0.001***	16 [13–20]	27 [24–30]	39 [35–41]	** < *0.001***
HADS-A (median (IQR))	3 [2–5]	5 [3–7]	***0.001***	4 [1–6]	6 [4–8]	7 [4–10]	***0.001***^***c,d,e,f***^
HADS-D (median (IQR))	1 [0–2]	3 [1–5]	** < *0.001***	1 [0–2]	4 [2–5]	5 [2–8]	** < *0.001***^***c,d,e,f***^

Normally distributed variables are summarized by mean ± standard deviation, while non-normally distributed variables are summarized by median (interquartile range)

*HC* healthy controls, *pwMS* people with multiple sclerosis, *F* female, *M* male, *CIS* clinically isolated syndrome, *RRMS* relapsing–remitting multiple sclerosis, *PMS* progressive multiple sclerosis, *EDSS* Expanded disability status scale, *IQR *interquartile range, *FSMC *fatigue scale of motor and cognitive function, *HADS* Hospital Anxiety and Depression Scale, *HADS-A* anxiety subscale, *HADS-D* depression subscale. Significant *p*-values are marked in bold.

^a^*p* values were compared between HC and groups of pwMS, or between groups of pwMS in case of MS-specific variables

^b^Significant contrast between HC and non-fatigued pwMS

^c^Significant contrast between HC and mildly/moderately fatigued pwMS

^d^Significant contrast between HC and severely fatigued pwMS

^e^Significant contrast between non-fatigued and mildly/moderately fatigued pwMS

^f^Significant contrast between non-fatigued and severely fatigued pwMS

^g^Significant contrast between mildly/moderately fatigued pwMS and severely fatigued pwMS

Distributions of MS phenotypes were significantly different between groups of pwMS based on fatigue (*p* = 0.027). Mildly/moderately fatigued pwMS and severely fatigued pwMS showed higher EDSS scores than non-fatigued pwMS (both *p* < 0.05). Compared to non-fatigued pwMS, mildly/moderately fatigued pwMS showed higher disease duration (*p* = 0.009). Lesion volume was higher in severely fatigued pwMS compared to non-fatigued pwMS (*p* < 0.05).

In our sample, 85 pwMS (39.2%) showed no cognitive fatigue (FSMC_cognitive_ < 22), 93 (42.9%) showed mild/moderate cognitive fatigue (22 ≤ FSMC_cognitive_ < 34), and 39 (18.0%) showed severe cognitive fatigue (FSMC_cognitive_ ≥ 34). Concerning motor fatigue, 87 pwMS (40.1%) showed no motor fatigue (FSMC_motor_ < 22), 68 (31.3%) showed mild/moderate motor fatigue (22 ≤ FSMC_motor_ < 32), and 62 (28.6%) showed severe motor fatigue (FSMC_motor_ ≥ 32). The majority of pwMS showed overlap regarding severity of cognitive and motor fatigue, with, respectively, only 5 (2.3%) pwMS showing mild/moderate cognitive fatigue and 1 (0.5%) pwMS showing severe cognitive fatigue in the absence of motor fatigue, and respectively, 3 (1.4%) pwMS showing mild/moderate motor fatigue and 6 (2.8%) pwMS showing severe motor fatigue in the absence of cognitive fatigue.

Table 2 shows the associations of total, cognitive, and motor fatigue with demographic and clinical variables.

**Table 2:** Associations of fatigue with demographics, clinical variables, anxiety and depression per group

	HC (*n* = *60)*			pwMS (*n* = *217)*		
	Total fatigue	Cognitive fatigue	Motor fatigue	Total fatigue	Cognitive fatigue	Motor fatigue
Sex (W)	473	472	479	6364.50	6437.50	6146.00
Age (ρ)	0.11	0.11	0.06	0.09	− 0.01	0.17*
Years of education (ρ)	0.10	0.13	0.03	− 0.14*	− 0.10	− 0.17*
MS type (χ^2^)	*NA*	*NA*	*NA*	9.34*	6.80	19.94***
EDSS (ρ)	*NA*	*NA*	*NA*	0.37***	0.21**	0.47***
Lesion load (ρ)	*NA*	*NA*	*NA*	0.09	0.01	0.15*
Disease duration (ρ)	*NA*	*NA*	*NA*	4.97	6.47*	2.86
Treatment group (W)	*NA*	*NA*	*NA*	0.14*	0.11	0.16*
HADS-A (ρ)	0.46***	0.49***	0.32*	0.39***	0.45***	0.28***
HADS-D (ρ)	0.32*	0.28*	0.24	0.59***	0.55***	0.55***

Shown are the statistical parameters reflecting the strength of the relationship between total, motor, and cognitive fatigue and demographics and neuropsychological variables. All associations were tested using non-parametric tests. Significant *p*-values are marked in bold. *p < 0.05; **p < 0.01; ***p < *0.001.*

*HC* healthy control, *pwMS* people with multiple sclerosis, *W* Wilcoxon Signed Rank Test Statistic, *ρ* Spearman’s rank correlation coefficient, χ^2^ Kruskal–Wallis Chi-squared statistic, *EDSS* Expanded Disability Status Scale, *HADS-A* Hospital Anxiety and Depression Scale-Anxiety Subscale, *HADS-D* Hospital Anxiety and Depression Scale-Depression Subscale

### Total fatigue

Table 3 depicts all group comparisons of total fatigue, which were all corrected for age, sex, and years of education. For most analyses showing group differences, age and sex were significant covariates, whereas years of education were not.

**Table 3:** Differences between healthy controls and people with MS grouped by fatigue severity

	*HC (n* = *60)*	*Non-fatigued pwMS (n* = *87)*	*Mildly/moderately fatigued pwMS (n* = *77)*	*Severely fatigued pwMS (n* = *53)*	***F/χ***^***2***^	***p***_***uncorr***_	***p***^***a***^
Normalized gray matter volumes (cm^3^)							
Thalamic volume	21.831 ± 1.499	20.501 ± 2.279	20.152 ± 2.264	19.809 ± 1.830	21.704	** < *0.001***	** < *0.001***^***c,d,e,g***^
Basal ganglia volume	27.500 ± 2.116	26.174 ± 2.582	25.980 ± 2.320	25.432 ± 2.441	18.443	** < *0.001***	** < *0.001***^***c,d,e***^
Cortical grey matter	790.242 ± 66.625	747.236 ± 66.004	725.406 ± 68.746	728.705 ± 72.204	38.332	** < *0.001***	** < *0.001***^***c,d,e,f***^
5-HT_1a_ circuit volume	22.901 ± 1.988	21.801 ± 2.264	21.200 ± 2.012	20.980 ± 2.139	13.740	** < *0.001***	** < *0.001***^***c,d,e,g***^
5-HT_2a_ circuit volume	32.871 ± 5.201	31.638 ± 3.903	30.553 ± 4.511	31.423 ± 4.811	17.006	** < *0.001***	** < *0.001***^***d***^
5-HTT circuit volume	47.231 ± 3.116	44.761 ± 4.405	43.945 ± 3.968	43.413 ± 3.890	20.401	** < *0.001***	** < *0.001***^***c,d,e,g***^
D_1_ circuit volume	23.919 ± 1.965	22.578 ± 2.328	22.517 ± 2.175	21.929 ± 2.259	21.195	** < *0.001***	** < *0.001***^***c,d,e***^
D_2_/DAT circuit volume	28.794 ± 2.218	27.371 ± 2.739	27.142 ± 2.484	26.582 ± 2.556	19.152	** < *0.001***	** < *0.001***^***c,d,e***^
NAT circuit volume	47.981 ± 4.292	44.123 ± 5.559	42.803 ± 5.180	42.809 ± 5.372	32.087	** < *0.001***	** < *0.001***^***c,d,e***^
Static functional connectivity							
Within 5-HT_1a_ circuit FC	0.281 ± 0.125	0.257 ± 0.125	0.234 ± 0.099	0.253 ± 0.12	2.632	***0.050***	0.101
Within 5-HT_2a_ circuit FC	0.384 ± 0.119	0.365 ± 0.109	0.341 ± 0.097	0.361 ± 0.118	3.141	***0.026***	0.077
Within 5-HTT circuit FC	0.171 ± 0.082	0.155 ± 0.051	0.153 ± 0.052	0.151 ± 0.054	3.842	***0.010***	***0.041***
Within D_1_ circuit FC	0.263 ± 0.119	0.245 ± 0.074	0.24 ± 0.09	0.245 ± 0.093	4.732	***0.003***	***0.016***
Within D_2_/DAT circuit FC	0.210 ± 0.092	0.191 ± 0.053	0.191 ± 0.065	0.192 ± 0.068	5.032	***0.002***	***0.012***
Within NAT circuit FC	0.533 ± 0.198	0.539 ± 0.228	0.509 ± 0.208	0.495 ± 0.235	1.544	0.204	0.204
Dynamic reconfiguration							
Global flexibility	0.207 ± 0.022	0.209 ± 0.028	0.215 ± 0.030	0.220 ± 0.033	7.441	** < *0.001***	***0.001***^***e,g***^
Post-hoc: Global cohesion	0.182 ± 0.022	0.185 ± 0.028	0.190 ± 0.029	0.196 ± 0.033	7.222	** < *0.001***	** < *0.001***^***e,g***^
Post-hoc: Global disjointedness	0.024 ± 0.003	0.025 ± 0.003	0.025 ± 0.003	0.025 ± 0.003	0.868	0.458	0.842
Global promiscuity	0.735 ± 0.057	0.746 ± 0.052	0.754 ± 0.052	0.762 ± 0.052	4.453	***0.005***	***0.032***^***e***^
5-HT_1a_ flexibility	0.281 ± 0.034	0.286 ± 0.044	0.293 ± 0.041	0.298 ± 0.048	3.988	***0.008***	***0.050***^***e***^
Post-hoc: 5-HT_1a_ cohesion	0.243 ± 0.032	0.247 ± 0.040	0.253 ± 0.038	0.260 ± 0.046	5.002	***0.002***	***0.013***^***e***^
Post-hoc: 5-HT_1a_ disjointedness	0.038 ± 0.009	0.039 ± 0.011	0.039 ± 0.010	0.038 ± 0.009	0.461	0.710	0.842
5-HT_2a_ flexibility	0.195 ± 0.031	0.195 ± 0.037	0.202 ± 0.042	0.198 ± 0.035	2.006	0.113	0.205
5-HTT flexibility	0.328 ± 0.036	0.328 ± 0.037	0.333 ± 0.033	0.337 ± 0.032	3.154	***0.025***	0.106
D_1_ flexibility	0.322 ± 0.039	0.323 ± 0.040	0.328 ± 0.040	0.325 ± 0.038	2.584	0.054	0.161
D_2_/DAT flexibility	0.328 ± 0.037	0.329 ± 0.037	0.334 ± 0.036	0.334 ± 0.031	3.117	***0.027***	0.106
NAT flexibility	0.188 ± 0.055	0.182 ± 0.062	0.191 ± 0.065	0.196 ± 0.071	1.540	0.205	0.205
5-HT_1a_ promiscuity^*b*^	0.86 [0.84–0.88]	0.86 [0.84–0.88]	0.86 [0.84–0.88]	0.86 [0.84–0.88]	3.720	0.293	0.572
5-HT_2a_ promiscuity^*b*^	0.74 [0.70–0.79]	0.77 [0.72–0.8]	0.78 [0.75–0.81]	0.76 [0.74–0.8]	9.913	***0.019***	0.116
5-HTT promiscuity^*b*^	0.87 [0.86–0.88]	0.88 [0.86–0.88]	0.88 [0.86–0.88]	0.88 [0.86–0.88]	2.339	0.505	0.572
D_1_ promiscuity^*b*^	0.88 [0.85–0.88]	0.88 [0.85–0.88]	0.88 [0.85–0.88]	0.88 [0.85–0.88]	2.166	0.539	0.572
D_2_/DAT promiscuity^*b*^	0.88 [0.86–0.88]	0.88 [0.86–0.88]	0.88 [0.86–0.88]	0.88 [0.86–0.88]	2.001	0.572	0.572
NAT promiscuity^*b*^	0.71 [0.61–0.78]	0.74 [0.62–0.81]	0.74 [0.64–0.83]	0.79 [0.60–0.84]	6.566	0.087	0.436

Significant *p*-values are marked in bold. *HC* healthy controls, *pwMS* people with multiple sclerosis, *5-HT*_*1a*_^*5-HT*^serotonin-1a receptor, *5-HT*_*2a*_ serotonin-2a receptor, *5-HTT* serotonin transporter, *D*_*1*_ dopamine-1 receptor, *D*_*2*_ dopamine-2 receptor, *DAT* dopamine transporter, *NAT* noradrenalin transporter, *FC* Functional Connectivity

^a^Reported omnibus p-values were corrected for multiple comparisons per circuit using Benjamini–Hochberg correction

^b^Non-normally distributed; reported statistic is Kruskal–Wallis chi-squared

^c^Significant contrast between HC and non-fatigued pwMS

^d^Significant contrast between HC and mildly/moderately fatigued pwMS

^e^Significant contrast between HC and severely fatigued pwMS

^f^Significant contrast between non-fatigued and mildly/moderately fatigued pwMS

^g^Significant contrast between non-fatigued and severely fatigued pwMS

^h^Significant contrast between mildly/moderately fatigued pwMS and severely fatigued pwMS

#### Gray matter volumes

Thalamic volume was lower in all pwMS groups than in HC (all *p* < 0.001), and lower in severely fatigued pwMS than in non-fatigued pwMS (*p* = 0.043). Basal ganglia volume was lower in all pwMS groups than in HC (all *p* < 0.001). Cortical GM volume was lower in all pwMS groups than in HC (all *p* < 0.001), and in mildly/moderately fatigued pwMS compared to non-fatigued pwMS (*p* = 0.018).

5-HT_1a_ circuit GM volume was lower in mildly/moderately and severely fatigued pwMS groups than in HC (both *p* < 0.001), in non-fatigued pwMS compared to HC (*p* = 0.024), and in severely fatigued pwMS compared to non-fatigued pwMS (*p* = 0.020). 5-HT_2a_ circuit GM volume was lower in mildly/moderately fatigued pwMS compared to HC (*p* = 0.001). 5-HTT circuit GM volume was lower in all pwMS groups than in HC (all *p* < 0.001), and in severely fatigued pwMS compared to non-fatigued pwMS (*p* = 0.042). GM volumes of D_1_, D_2_/DAT, and NAT circuits were lower in all pwMS groups compared to HC (all *p* < 0.001).

#### Static functional connectivity

Functional connectivity within monoaminergic circuits showed significant group differences for the 5-HTT (*p*_*adj*_ = 0.041), D_1_ (*p*_*adj*_ = 0.016), and D_2_/DAT circuits (*p*_*adj*_ = 0.012), however, no significant pairwise contrasts were observed (all *p* > 0.05), possibly due to the strong effect of the covariates age and sex. However, descriptive values show comparably higher scores for the HC, with lower mean values for pwMS with no, mild/moderate, or severe fatigue.

#### Reconfiguration dynamics

**Fig. 3:**
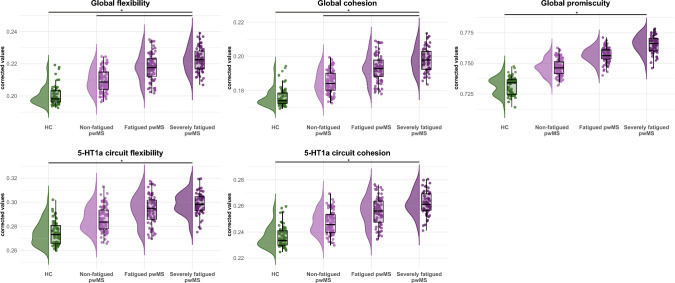
Significant group differences in dynamic reconfiguration of the whole brain and monoaminergic circuits. Shown are figures of dynamic reconfiguration variables compared between groups. Values on the y-axis are residuals of the model after correction for sex, age, and years of education. *Significant group difference. *HC* healthy controls, *pwMS* people with multiple sclerosis, *5-HT*_*1a*_ serotonin-1a receptor

Global flexibility was higher in severely fatigued pwMS than in HC (*p* = 0.016) and in non-fatigued pwMS (*p* = 0.025; Fig. 3). Post-hoc analyses showed higher global cohesion in severely fatigued pwMS than in HC (*p* = 0.017) and in non-fatigued pwMS (*p* = 0.022; Fig. 3).

Global promiscuity was higher in severely fatigued pwMS than in HC (*p* = 0.014).

Concerning monoaminergic circuits, we observed higher 5-HT_1a_ circuit flexibility in severely fatigued pwMS than in HC (*p* = 0.048; Fig. 3. Post-hoc analysis showed higher 5-HT_1a_ circuit cohesion in severely fatigued pwMS than in HC (*p* = 0.031). No other circuit-specific measures of reconfiguration dynamics showed any group differences.

### Sensitivity analysis based on relapsing–remitting MS only

To verify that our results were not only driven by the most progressive pwMS in our sample, we re-ran our analyses using relapsing–remitting MS only. In doing so, we reproduced all reported group differences on total fatigue, and found additional significant pairwise contrasts between mildly/moderately fatigued pwMS and HC for global flexibility (*p* = 0.025), global cohesion (*p* = 0.033) and global promiscuity (*p* = 0.031). Results are reported in Table S3.

### Exploratory analyses on associations with cognitive or motor fatigue

Post-hoc, we estimated whether group differences in total fatigue were rather driven by either cognitive fatigue or motor fatigue (Table S4).

As in the analyses with grouping based on total fatigue scores, while comparing based on either cognitive or motor fatigue, within-circuit FC of the 5-HTT, D_1_, and D_2_/DAT circuits showed significant group differences with the same patterns; however, no significant pairwise contrasts were observed after correcting for multiple comparisons (all *p* > 0.05; Table S4).

Concerning reconfiguration dynamics, uncorrected values show patterns in line with total fatigue for both cognitive and motor fatigue. However, after correction for multiple comparisons, the difference in global flexibility and global cohesion between severely fatigued pwMS and non-fatigued pwMS remained significant only when comparing groups based on motor fatigue (*p*_*flexibility*_ = 0.049; *p*_*cohesion*_ = 0.048). Similarly, the difference in 5-HT_1a_ circuit flexibility and cohesion between severely fatigued pwMS and HC remained significant only when comparing groups based on motor fatigue (*p*_*flexibility*_ = 0.045; *p*_*cohesion*_ = 0.037).

No further variables showed incongruent patterns of group differences between cognitive and motor fatigue.

## Discussion

This study investigated if and how brain functional dynamics in monoaminergic circuits are involved in MS-related fatigue. In line with our hypotheses, we observed that higher network connectivity dynamics were associated with MS-related fatigue. Specifically, we observed more frequent and more synchronous network switching in severely fatigued pwMS compared to non-fatigued pwMS and HC. This was observed globally and in inhibitory (5-HT_1a_) serotonergic receptor circuits. In our sample, the majority of patients showed an overlap of cognitive and motor fatigue, leading to similar patterns of functional connectivity. However, significant differences between non-fatigued and severely fatigued pwMS were only observed related to motor fatigue, probably due to the higher prevalence of severely motor fatigued pwMS (*n* = 62) compared to severely cognitively fatigued pwMS (*n* = 39).

Globally, we observed higher flexibility (i.e. more switches between resting-state networks [RSNs]) in severely fatigued pwMS than in non-fatigued pwMS and HC. Since switching between connectivity patterns requires more energy in pwMS [12], higher flexibility may also contribute to MS-related fatigue through increasing metabolic demand in pwMS.

Furthermore, flexibility is known to be higher while moving through developmental stages, and often co-occurs with relatively weak connection strength. This specific combination is suggested to enable the brain to achieve many difficult-to-reach states, reflecting a capacity to adapt to novel situations [35]. Thus, in MS, increased flexibility may be a plausible response to MS-related pathology. However, while higher flexibility is associated with adaptive behavior, excessive flexibility has been reported as maladaptive across contexts [10]. For instance, just as excessive visual system flexibility in infants is associated with slower passing of visual milestones [36], higher flexibility in pwMS may also hinder efficient brain function. For instance, we know that in healthy subjects, statistical variability in flexibility is explained by state-like fatigue [37]. Therefore, permanently elevated flexibility may result in a more trait-like continuous experience of fatigue in pwMS, which might render the brain better able to overcome MS-induced network disconnection, at the cost of fatigue.

Additionally, individual regions were more likely to make the same network switch as other regions at the same time (cohesion) in severely fatigued pwMS than in non-fatigued pwMS and HC. In healthy subjects, higher network cohesion during learning was associated with faster rates of learning. Possibly, because cohesion signals “linked” regions that associate with the same large-scale networks at the same time, higher cohesion might reflect successful predictive processing, and thus successful learning, by higher-order brain regions [10, 34]. Similarly, MS pathology might provoke a coordinated network response from higher-order brain areas, which, in order to effectively reorganize, link their dynamic connectivity patterns, reflected in higher cohesion. Such a phenomenon would align well with the concept of brain reserve in MS, and might come at the cost of a subjective experience of fatigue [38]. Alternatively, in previous work, higher cohesion was interpreted to signal higher viscosity of the network [11], meaning that brain regions lose the ability to switch RSNs independently and instead are ‘pulled into’ switches of their connectivity neighbors. In this scenario, regions are less autonomous in steering their dynamic behavior, resulting in a loss of the dynamics in individual regions that support effective brain function. This would then explain why higher cohesion seems to be maladaptive in the context of MS [11].

Concerning monoaminergic dynamics, regions of the 5-HT_1a_ circuit (inhibitory 5-HT receptor) also switched interactions with RSNs more frequently (flexibility) and more often synchronously with other regions (cohesion) in severely fatigued pwMS compared to non-fatigued pwMS and HC. Again, with regions switching networks more often and with more regions at the same time, metabolic demand is likely increased [12]. Since serotonergic signaling is related to subjective energy levels and mood disorders, network disruption in serotonergic circuits may indeed partially underlie MS-related fatigue. Excessive unstable serotonergic signaling may lead to desensitization of the 5-HT_1a_ receptor, which not only contributes directly to fatigue,[20] but also might disinhibit the rest of the serotonergic system. Beyond potentially fatigue-inducing network aberrations [5], this may result in excess serotonin in the synaptic cleft, which has also been linked to fatigue [39].

Concerning static FC, we observed significant group differences for within-circuit FC of the serotonin transporter circuit and all dopaminergic circuits; however, we found no significant pairwise group differences. This might be because of strong effects of sex and age, denoting an interaction between demographics and FC patterns of monoaminergic circuits. Descriptive patterns, however, show that static FC is higher in HC compared to all pwMS, and decreases with rising fatigue. This aligns with the dynamic findings, as more unstable dynamic FC may lead to decreased static FC in individual networks.[10] Nevertheless, we did not replicate previously reported static FC patterns linked to MS-related fatigue [7, 13], likely because of methodological differences in granularity and definition of monoaminergic circuits. All in all, network dynamics might be more sensitive compared to static FC patterns when exploring MS-related fatigue.

Regarding fatigue subtypes, we expected to observe noradrenergic and dopaminergic effects rather related to cognitive fatigue. However, given that the majority of patients in our sample showed an overlap between cognitive and motor fatigue, with only 4.2% of patients showing distinct motor fatigue, we were not able to identify specific patterns of FC related to either cognitive or motor fatigue. As shown in the supplement, FC showed similar patterns related to both cognitive and motor fatigue, but probably due to the higher number of patients with severe motor fatigue (*n* = 62) compared to severe cognitive fatigue *(n* = 39), significant differences were only observed for the motor fatigue group. Larger multicenter samples are needed to further explore the distinct mechanisms of motor vs cognitive fatigue.

Our study is the first to investigate the dynamic properties of monoaminergic circuits in the context of MS-related fatigue. As we were specifically interested in fatigue as associated with MS, we excluded any participants reporting clinically significant depression, which is known to be strongly associated with fatigue.[40] Further, our sensitivity analyses including relapsing–remitting pwMS only revealed that our findings were not merely driven by the most progressive cases. Finally, global reconfiguration variables were comparable to those reported for a similar cohort exploring cognitive dysfunction in MS [11], but promiscuity was slightly higher, likely due to the higher number of sliding windows used here.

Some limitations of the current work should be noted. First, despite our data-driven cut-off to determine which regions to include in each monoaminergic circuit, for serotonergic and noradrenergic circuits, there was no clear change point, and so a cut-off was established. Second, as no noradrenergic receptor maps were publicly available, potential relationships of noradrenergic receptor circuits with fatigue remain obscure. Lastly, functional images did not cover the cerebellum and therefore incompletely captured networks and circuits of interest. Future studies in the context of monoaminergic connectivity should consider estimating non-linear relationships with behavioral outcomes, and disentangling inhibitory and excitatory connections.

Overall, we found that severely fatigued pwMS show more unstable functional dynamics both globally and in inhibitory serotonergic circuits. Combined with overall lower serotonergic connectivity strength, this may depict a failure of focused (i.e., stable) communication patterns, which likely induces increased energy expenditure leading to fatigue. This theory aligns with previous evidence of more chaotic local connectivity dynamics in pwMS [11]. Importantly, beyond atypical and unstable network recruitment, MS pathology may also induce fatigue by desensitizing inhibitory serotonergic receptors, which may disrupt the serotonergic system as a whole and thus contribute to unstable serotonergic network recruitment. Future longitudinal studies should confirm this mechanistic theory of MS-related fatigue to advance the development of therapeutic approaches targeted at alleviating fatigue in pwMS.

## Supplementary Information

Below is the link to the electronic supplementary material.

Supplementary file1 (DOCX 50 KB)

## Data Availability

Data collected as part of this study are available only upon reasonable request. Scripts for pre- and post-processing of MR images are openly available through https://github.com/marloesbet/fatigue_dynamics.
