# Supplementary material for: Functional connectivity dynamics of monoaminergic circuits related to fatigue in multiple sclerosis
Source: J Neurol. 2026 Jun 2;273(6):354. doi: 10.1007/s00415-026-13876-0 (PMC13230267; doi:10.1007/s00415-026-13876-0)
Supplement: Supplementary file 1 — Supplementary file1 (DOCX 50 KB) [file 415_2026_13876_MOESM1_ESM.docx]

**Table S1.** Receptor and transporter density atlases based on positron emission tomography.

| **Map** | **Tracer** | **Measure** | ***N*** | **Age** | **Res.** | **Reference** |
| --- | --- | --- | --- | --- | --- | --- |
| 5-HT_1a_ (R) | [^11^C]WAY-100635 | *BP_ND_* | 35 | 26.30±5.20 | 3mm | Savli et al., 2012 [23] |
| 5-HT_2a_ (R) | [^11^C]Cimbi-36 | *B_max_* | 29 | 22.60±2.70 | 1mm | Beliveau et al., 2017 [24] |
| 5-HTT (T) | [^11^C]DASB | *B_max_* | 100 | 25.10±5.80 | 1mm | Beliveau et al., 2017 [24] |
| D_1_ (R) | [^11^C]SCH23390 | *BP_ND_* | 13 | 33.00±13.00 | 3mm | Kaller et al., 2017 [25] |
| D_2_ (R) | [^11^C]Raclopride | *BP_ND_* | 156 | 27.76±9.44 | 2mm | Malén et al., 2022 [26] |
| DAT (T) | [^11^C]FPCIT (SPECT) | *SUVR* | 174 | 61.00±11.00 | 3mm | Dukart et al., 2018 [27] |
| NAT (T) | S,S-[^11^C]O-MRB | *BP_ND_* | 77 | 33.40±9.17 | 1mm | Ding et al., 2010 [28] |

All atlas maps were obtained from neuromaps (*https://github.com/netneurolab/neuromaps*) in MNI152 standard space. *Res.* Resolution, *5-HT* Serotonin, *D/DA* Dopamine, *NA* Noradrenalin, *R* Receptor, *T* Transporter, *SPECT* Singe Photon Emission Computed Tomography, *BP_ND_* Binding Potential-Non Displaceable, *B_max_* Maximum Binding, *SUVR* Standard Uptake Value Ratio, *MRB* Methylreboxetine

**Table S2.** Regions included per monoaminergic receptor/transporter circuit.

| **5-HT_1a_** | **5-HT_2a_** | **5-HTT** | **D_1_** | **D_2_/DAT (same set of regions)** | **NAT** |
| --- | --- | --- | --- | --- | --- |
| **109** Rostral area 35/36 L (LN) | **23** Ventrolateral area 8 L (DMN) | **117** Temporal agranular insular cortex L (LN) | **212** Caudate L (DGM) | **212** Caudate L (DGM) | **57** Upper limb region L (SMN) |
| **110** Rostral area R 35/36 (LN) | **37** Opercular area 44 L (VAN) | **118** Temporal agranular insular cortex R (LN) | **213** Putamen L (DGM) | **213** Putamen L (DGM) | **59** Trunk region L (SMN) |
| **111** Caudal area 35/36 L (LN) | **39** Ventral area 44 L (DMN) | **211** Thalamus L (DGM) | **217** Accumbens L (DGM) | **214** Pallidum L (DGM) | **60** Trunk region R (SMN) |
| **112** Caudal area 35/36 R (LN) | **81** Caudal area 21 L (DMN) | **213** Putamen L (DGM) | **219** Caudate R (DGM) | **217** Accumbens L (DGM) | **67** Lower limb region L (SMN) |
| **113** Lateral posterior parahippocampal gyrus L (DMN) | **82** Caudal area 21 R (DMN) | **214** Pallidum L (DGM) | **220** Putamen R (DGM) | **219** Caudate R (DGM) | **68** Lower limb region R (SMN) |
| **114** Lateral posterior parahippocampal gyrus R (VN) | **85** Dorsolateral area 37 L (DAN) | **216** Amygdala L (DGM) | **224** Accumbens R (DGM) | **220** Putamen R (DGM) | **72** Area 41/42 R (SMN) |
| **117** Temporal agranular insular cortex L (LN) | **86** Dorsolateral area 37 R (DAN) | **217** Accumbens L (DGM) |  | **221** Pallidum R (DGM) | **155** Area 1/2/3 (upper limb, head and face region) L (SMN) |
| **166** Ventral agranular insula R (VAN) | **87** Anterior superior temporal sulcus L (DMN) | **218** Thalamus R (DGM) |  | **224** Accumbens R (DGM) | **156** Area 1/2/3 (upper limb, head and face region) R (SMN) |
| **169** Ventral dysgranular and granular insula L (VAN) | **88** Anterior superior temporal sulcus R (DMN) | **220** Putamen R (DGM) |  |  | **161** Area 1/2/3 (trunk region) L (SMN) |
| **170** Ventral dysgranular and granular insula R (VAN) | **97** Ventrolateral area 37 L (DAN) | **221** Pallidum R (DGM) |  |  | **162** Area 1/2/3 (trunk region) R (SMN) |
| **215** Hippocampus L (DGM) | **99** Caudolateral area 20 L (FPN) | **223** Amygdala R (DGM) |  |  | **211** Thalamus L (DGM) |
| **222** Hippocampus R (DGM) | **123** Caudoposterior superior temporal sulcus L (DMN) | **224** Accumbens R (DGM) |  |  | **218** Thalamus R (DGM) |

**Table S3.** Differences between healthy controls and people with relapsing-remitting MS grouped by fatigue severity.

|  | ***HC (n=60)*** | ***Non-fatigued pwMS (n=85)*** | ***Mildly/moderately fatigued pwMS (n=65)*** | ***Severely fatigued pwMS (n=44)*** | ***F/χ^2^*** | ***p*** | ***p^a^*** |
| --- | --- | --- | --- | --- | --- | --- | --- |
| *Normalized Grey Matter Volumes (cm^3^)* | | | | | | | |
| Thalamic volume | 21.948 ± 1.499 | 20.569 ± 2.224 | 20.208 ± 2.105 | 19.779 ± 1.881 | 20.090 | ***<0.001*** | ***<0.001^c,d,e,g^*** |
| Basal ganglia volume | 27.582 ± 2.116 | 26.264 ± 2.562 | 26.209 ± 2.268 | 25.529 ± 2.476 | 15.061 | ***<0.001*** | ***<0.001^c,d,e^*** |
| Cortical grey matter | 794.886 ± 66.625 | 750.334 ± 64.662 | 730.230 ± 62.894 | 731.382 ± 71.691 | 31.539 | ***<0.001*** | ***<0.001^c,d,e,f^*** |
| 5-HT_1a_ circuit volume | 22.927 ± 1.988 | 21.897 ± 2.158 | 21.303 ± 2.013 | 20.979 ± 2.105 | 11.959 | ***<0.001*** | ***<0.001^c,d,e,g^*** |
| 5-HT_2a_ circuit volume | 33.243 ± 5.201 | 31.827 ± 3.828 | 30.639 ± 4.069 | 31.348 ± 4.363 | 14.128 | ***<0.001*** | ***<0.001^c,d,e^*** |
| 5-HTT circuit volume | 47.455 ± 3.116 | 44.883 ± 4.340 | 44.210 ± 3.942 | 43.517 ± 3.934 | 16.772 | ***<0.001*** | ***<0.001^c,d,e^*** |
| D_1_ circuit volume | 24.003 ± 1.965 | 22.656 ± 2.307 | 22.745 ± 2.115 | 22.049 ± 2.268 | 17.511 | ***<0.001*** | ***<0.001^c,d,e^*** |
| D_2_/DAT circuit volume | 28.884 ± 2.218 | 27.462 ± 2.716 | 27.400 ± 2.418 | 26.704 ± 2.573 | 15.558 | ***<0.001*** | ***<0.001^c,d,e^*** |
| NAT circuit volume | 48.308 ± 4.292 | 44.278 ± 5.359 | 43.033 ± 4.801 | 42.883 ± 5.582 | 28.221 | ***<0.001*** | ***<0.001^c,d,e^*** |
| *Static functional connectivity* | | | | | | | |
| Within 5-HT_1a_ circuit FC | 0.284 ± 0.125 | 0.254 ± 0.123 | 0.231 ± 0.102 | 0.253 ± 0.12 | 2.550 | 0.056 | 0.067 |
| Within 5-HT_2a_ circuit FC | 0.39 ± 0.119 | 0.363 ± 0.109 | 0.344 ± 0.099 | 0.359 ± 0.113 | 2.605 | 0.052 | 0.067 |
| Within 5-HTT circuit FC | 0.173 ± 0.082 | 0.155 ± 0.051 | 0.154 ± 0.054 | 0.15 ± 0.051 | 3.850 | ***0.010*** | ***0.020*** |
| Within D_1_ circuit FC | 0.267 ± 0.119 | 0.246 ± 0.074 | 0.239 ± 0.092 | 0.249 ± 0.094 | 4.276 | ***0.006*** | ***0.018*** |
| Within D_2_/DAT circuit FC | 0.213 ± 0.092 | 0.192 ± 0.053 | 0.192 ± 0.066 | 0.194 ± 0.067 | 4.592 | ***0.004*** | ***0.018*** |
| Within NAT circuit FC | 0.544 ± 0.198 | 0.533 ± 0.226 | 0.504 ± 0.216 | 0.488 ± 0.231 | 1.664 | 0.175 | 0.175 |
| *Dynamic reconfiguration* | | | | | | | |
| Global flexibility | 0.205 ± 0.022 | 0.208 ± 0.028 | 0.217 ± 0.028 | 0.22 ± 0.031 | 7.357 | ***<0.001*** | ***<0.001^d,e,g^*** |
| *Post-hoc: Global cohesion* | 0.18 ± 0.022 | 0.184 ± 0.028 | 0.192 ± 0.027 | 0.195 ± 0.031 | 7.113 | ***<0.001*** | ***<0.001^d,e,g^*** |
| *Post-hoc: Global disjointedness* | 0.024 ± 0.003 | 0.025 ± 0.003 | 0.025 ± 0.003 | 0.025 ± 0.003 | 0.941 | 0.421 | 0.421 |
| Global promiscuity | 0.731 ± 0.057 | 0.747 ± 0.052 | 0.754 ± 0.051 | 0.766 ± 0.051 | 4.596 | ***0.004*** | ***0.006^d,e,g^*** |
| 5-HT_1a_ flexibility | 0.279 ± 0.034 | 0.285 ± 0.045 | 0.292 ± 0.04 | 0.297 ± 0.045 | 3.427 | ***0.018*** | ***0.022^e^*** |
| *Post-hoc: 5-HT_1a_ cohesion* | 0.241 ± 0.032 | 0.246 ± 0.04 | 0.253 ± 0.036 | 0.26 ± 0.043 | 4.635 | ***0.004*** | ***0.006^e^*** |
| *Post-hoc: 5-HT_1a_ disjointedness* | 0.038 ± 0.009 | 0.039 ± 0.011 | 0.039 ± 0.01 | 0.037 ± 0.008 | 1.113 | 0.344 | 0.344 |
| 5-HT_2a_ flexibility | 0.193 ± 0.031 | 0.193 ± 0.036 | 0.202 ± 0.04 | 0.196 ± 0.032 | 1.956 | 0.121 | 0.182 |
| 5-HTT flexibility | 0.326 ± 0.036 | 0.328 ± 0.037 | 0.334 ± 0.034 | 0.338 ± 0.032 | 2.999 | ***0.031*** | 0.108 |
| D_1_ flexibility | 0.321 ± 0.039 | 0.322 ± 0.041 | 0.329 ± 0.041 | 0.324 ± 0.038 | 2.219 | 0.087 | 0.174 |
| D_2_/DAT flexibility | 0.326 ± 0.037 | 0.329 ± 0.037 | 0.334 ± 0.037 | 0.335 ± 0.032 | 2.897 | ***0.036*** | 0.108 |
| NAT flexibility | 0.185 ± 0.055 | 0.183 ± 0.062 | 0.196 ± 0.067 | 0.195 ± 0.07 | 1.707 | 0.166 | 0.199 |
| 5-HT_1a_ promiscuity*^b^* | 0.86 [0.84-0.88] | 0.86 [0.84-0.88] | 0.86 [0.84-0.88] | 0.86 [0.84-0.88] | 3.380 | 0.337 | 0.500 |
| 5-HT_2a_ promiscuity*^b^* | 0.74 [0.7-0.79] | 0.77 [0.72-0.8] | 0.78 [0.75-0.8] | 0.77 [0.74-0.81] | 9.494 | ***0.023*** | 0.138 |
| 5-HTT promiscuity*^b^* | 0.87 [0.86-0.88] | 0.88 [0.86-0.88] | 0.88 [0.86-0.88] | 0.88 [0.86-0.88] | 2.891 | 0.409 | 0.500 |
| D_1_ promiscuity*^b^* | 0.88 [0.85-0.88] | 0.88 [0.85-0.88] | 0.88 [0.85-0.88] | 0.88 [0.85-0.88] | 2.364 | 0.500 | 0.500 |
| D_2_/DAT promiscuity*^b^* | 0.88 [0.86-0.88] | 0.88 [0.86-0.88] | 0.88 [0.86-0.88] | 0.88 [0.86-0.88] | 2.452 | 0.484 | 0.500 |
| NAT promiscuity*^b^* | 0.71 [0.61-0.78] | 0.75 [0.62-0.81] | 0.74 [0.61-0.83] | 0.79 [0.65-0.85] | 7.698 | 0.053 | 0.159 |

Significant *p*-values are marked in bold.
*^a^*Reported omnibus p-values were corrected for multiple comparisons per circuit using Benjamini-Hochberg correction.
*^b^*Non-normally distributed; reported statistic is Kruskal-Wallis chi-squared.
*^c^*Significant contrast between HC and non-fatigued pwMS.
*^d^*Significant contrast between HC and mildly/moderately fatigued pwMS.
*^e^*Significant contrast between HC and severely fatigued pwMS.
*^f^*Significant contrast between non-fatigued and mildly/moderately fatigued pwMS.
*^g^*Significant contrast between non-fatigued and severely fatigued pwMS.
*^h^*Significant contrast between mildly/moderately fatigued pwMS and severely fatigued pwMS.
*HC* Healthy Controls, *pwMS* people with Multiple Sclerosis, *5-HT_1a_* Serotonin-1a Receptor, *5-HT_2a_* Serotonin-2a Receptor, *5-HTT* Serotonin Transporter, *D_1_* Dopamine-1 Receptor, *D_2_* Dopamine-2 Receptor, *DAT* Dopamine Transporter, *NAT* Noradrenalin Transporter

**Table S4.** Post-hoc pairwise group comparisons based on cognitive fatigue and motor fatigue.

| ***Outcome variable*** | ***HC (n=60)*** | ***Non-cognitive fatigued pwMS (n=85)*** | ***Mildly/moderately cognitive fatigued pwMS (n=93)*** | ***Severely cognitive fatigued pwMS (n=39)*** | ***F*** | ***p_adj_*** | ***Non-motor fatigued pwMS (n=87)*** | ***Mildly/moderately motor fatigued pwMS (n=68)*** | ***Severely motor fatigued pwMS (n=62)*** | ***F*** | ***p_adj_*** |
| --- | --- | --- | --- | --- | --- | --- | --- | --- | --- | --- | --- |
| Within D_1_ circuit FC | 0.263±0.119 | 0.245±0.075 | 0.241±0.089 | 0.243±0.097 | 4.477 | ***0.022*** | 0.24±0.074 | 0.248±0.094 | 0.241±0.089 | 4.744 | ***0.011*** |
| Within D_2_/DAT circuit FC | 0.209±0.092 | 0.193±0.053 | 0.190±0.064 | 0.192±0.070 | 4.942 | ***0.014*** | 0.188±0.049 | 0.198±0.071 | 0.19±0.064 | 5.344 | ***0.008*** |
| Within 5-HTT circuit FC | 0.171±0.082 | 0.155±0.050 | 0.152±0.054 | 0.153±0.054 | 3.700 | ***0.025*** | 0.152±0.045 | 0.16±0.062 | 0.148±0.049 | 4.428 | ***0.011*** |
| Global flexibility | 0.207±0.022 | 0.210±0.030 | 0.215±0.029 | 0.219±0.033 | 5.875 | ***0.005^f^*** | 0.21±0.031 | 0.214±0.026 | 0.22±0.033 | 7.280 | ***<0.001^f,h^*** |
| Global cohesion | 0.182±0.022 | 0.186±0.030 | 0.190±0.029 | 0.195±0.032 | 5.614 | ***0.007^f^*** | 0.186±0.03 | 0.189±0.026 | 0.195±0.032 | 6.999 | ***0.001^f,h^*** |
| Global promiscuity | 0.735±0.057 | 0.749±0.051 | 0.751±0.055 | 0.764±0.049 | 3.985 | ***0.025^f^*** | 0.749±0.054 | 0.752±0.049 | 0.759±0.054 | 3.770 | ***0.011^f^*** |
| 5-HT*_1a_* circuit flexibility | 0.281±0.034 | 0.288±0.046 | 0.292±0.041 | 0.295±0.049 | 2.978 | ***0.032*** | 0.287±0.044 | 0.291±0.039 | 0.298±0.049 | 4.154 | ***0.011^f^*** |
| 5-HT_1a_ circuit cohesion | 0.243±0.032 | 0.249±0.042 | 0.252±0.038 | 0.259±0.047 | 3.793 | ***0.025*** | 0.248±0.04 | 0.252±0.035 | 0.259±0.047 | 5.110 | ***0.009^f^*** |

Shown are F-statistics and p-values of performed ANCOVAs corrected for age, sex, and years of education, with predictor groups based on cognitive or motor fatigue. Only variables showing significant group differences based on total fatigue were tested. Reported *p*-values for main effects are adjusted for multiple comparisons using Benjamini-Hochberg correction per circuit. Significant *p*-values are marked in bold.
*^a^*Significant contrast between HC and severely fatigued pwMS. *^b^*Significant contrast between non-fatigued and severely fatigued pwMS. *HC* Healthy Controls, *pwMS* people with Multiple Sclerosis, *5-HT_1a_* Serotonin-1a Receptor, *5-HT_2a_* Serotonin-2a Receptor, *5-HTT* Serotonin Transporter, *D_1_* Dopamine-1 Receptor, *D_2_* Dopamine-2 Receptor, *DAT* Dopamine Transporter, *NAT* Noradrenalin Transporter, *FC* Functional Connectivity
